# [3 + 2] Cycloaddition reactions of thioisatin with thiazolidine-2-carboxylic acid: a versatile route to new heterocyclic scaffolds

**DOI:** 10.1186/2191-2858-1-6

**Published:** 2011-09-06

**Authors:** Sonali Verma, Johnson George, Saurabh Singh, Pushpa Pardasani, Ramchand Pardasani

**Affiliations:** 1 Department of Chemistry, University of Rajasthan, Jaipur - 302 055, INDIA

**Keywords:** Azabicycloadducts, 1,3-Dipolar cycloaddition reactions, AM1 Calculations, Thioisatin, Thiazolidine-2-carboxylic Acid

## Abstract

A facile synthesis of azabicycloadducts is described by 1,3-dipolar cycloaddition reactions of thioisatin with thiazolidine-2-carboxylic acid in the presence of various electron rich and electron deficient dipolarophiles. Theoritical calculations have been performed to study the regioselectivity of products. The geometrical and energetic properties have been analyzed for the different reactants, transition states and cycloadducts formed.

## Background

The construction of sophisticated molecules requires viable, selective and highly reliable reactions as potent synthetic tools [1-3]. The 1,3-dipolar cycloaddition [4-9] has also become one of the most important legation method in biology and material chemistry. Thioisatin derivatives [10] have received the attention of biochemists because of their therapeutic and biological activities. Similarly thiazolidine-2-carboxylic acid [11,12] exhibit strong antioxidant properties. Therefore any heterocyclic scaffold containing these two moieties might be expected to have considerably enhanced biological activities.

The 1,3-dipolar cycloaddition reaction of an azomethine ylide with an alkene leads to the formation of pyrrolidine [13,14] derivatives. Recently, we have reported the results on azomethine ylides derived from 9,10-phenanthrenequinone and some secondary cyclic α-amino acids with different dipolarophiles [15]. Herein we report the reactivity and regioselectivity of 1,3-dipolar cycloaddition reactions of azomethine ylides derived from benzo[b]thiophene-2,3-dione with thiazolidine-2-carboxylic acid in the presence of various acetylenic and ethylenic dipolarophiles. Besides synthetic work, a systematic and comprehensive theoretical study at Gaussian 03 [16] suite of programs has been carried out to address the mechanism as well as regio- and stereochemical course of the reactions.

## 2. Result and discussion

**Scheme 1:**
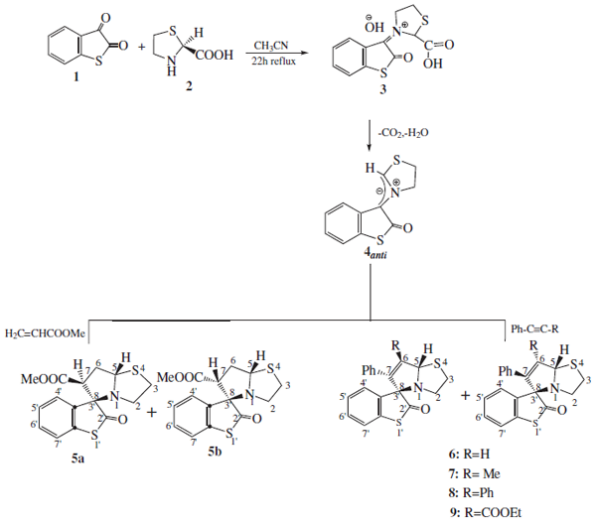
**Reaction of thioisatin with thiazolidine-2-carboxylic acid in presence of different dipolarophiles produced regioisomeric mixtures of cycloadducts**.

The reaction of thioisatin **1 **with thiazolidine-2-carboxylic **2 **acid was carried out in equimolar ratio in refluxing dry acetonitrile in the presence of diphenylacetylene as dipolarophile to afford a diastereomeric mixture of {(5R,8R)-spiro-6,7-diphenyl-1aza-4thia-bicyclo [3,3,0]-6-octene-8,3'}-benzo[b]thiophene-2'-one} **8 **as cis/trans isomers. Analogous reactions of thioisatin with other dipolarophiles *viz *methyl acrylate, phenylacetylene, phenylpropyne and ethyl phenyl propiolate produced diasteroisomeric mixtures of cycloadducts **5-9 **in 75%- 63% yield. The mechanism for the formation of the cycloadducts **5-9 **involve the initial formation of an iminium species **3 **followed by the loss of CO_2 _*via *stereospecific 1,3-cycloreversion [17] to azomethine ylides **4**. Subsequent [3 + 2] cycloaddition with various dipolarophiles then produce novel azabicycloadducts (Scheme 1).

The structure of all the cycloadducts has been ascertained from their spectral data. Thus the IR spectrum of a typical diphenylacetylene cycloadducts **8 **showed characteristic bands at 1715, 1420 and 690 for > C = O, C-N and C-S stretching vibrations respectively. Its ^1^H NMR spectrum showed a triplet at δ 2.51 (*J *= 2.7Hz) for 3H protons, another triplet at δ 2.60 (*J *= 3.0Hz) was associated with 2H protons, a singlet at δ 4.15 appeared for 5H and the aromatic protons resonated between δ 6.79- δ 8.12 ppm. Its ^13^C NMR spectrum showed a signal at 183.54 for C-2' carbonyl carbon, aromatic carbons appeared in the range δ 146.41-δ131.32 ppm, the olefenic carbons (C-6, C-7) resonated at δ 127.32 and 126.54, spiro carbon (C-8) was noticed at δ 86.23, C-5 at δ 46.72, C-2 at δ 45.43, and C-3 at 34.56 ppm respectively. Additional evidence was gathered from the mass spectrum of cycloadduct **8**. The molecular ion and the base peaks were present at m/z 413(32%) and 235(100%) respectively; another peak at m/z 385(39%) corresponded to [M-CO]^.+ ^whereas the peak at m/z 108(35%) was assigned to [C_6_H_4_S]^.+^.

### 2.1 Theoretical calculations: Regioselectivity of cycloadducts 5-9

The stereochemical course of the cycloaddition was examined by AM1 calculations. To calculate the relevant activation and stabilization energies, minimized geometries of the reactants, products and transition states are required. The molecular geometry of the simplest azomethine ylide (abbreviated as *am*y) **4 **derived from thioisatin and thiazolidine-2-carboxylic acid has been optimized on Gaussian 03 program at AM1 level.

**Figure 1:**
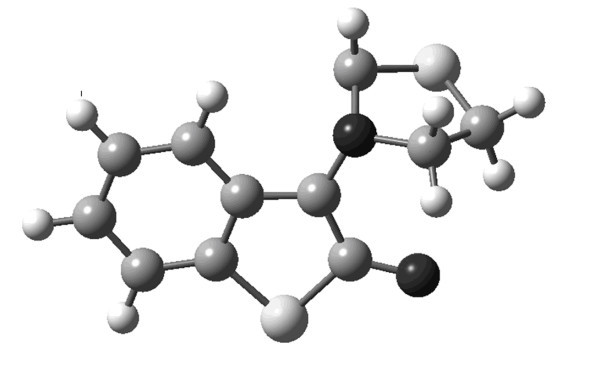
Optimized geometry of *amy *4_anti._

**Figure 2:**
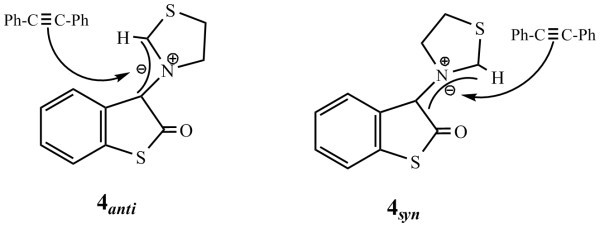
**Mode of attack of dipolarophile (diphenylacetylene) on *amy *4**.

Geometry optimization showed that *amy ***4 **has almost planar structure (Figure 1). Instead of having an envelope shape the thiazolidine ring is planar and lies in the same plane as that of thioisatin ring. It may exist in two isomeric forms, one in which > C = O group and C-H of the dipole are *syn *to each other, **4*****_syn _***, and the other in which these two groups are *anti*, **4*_anti _***(Figure 2).

**Figure 3:**
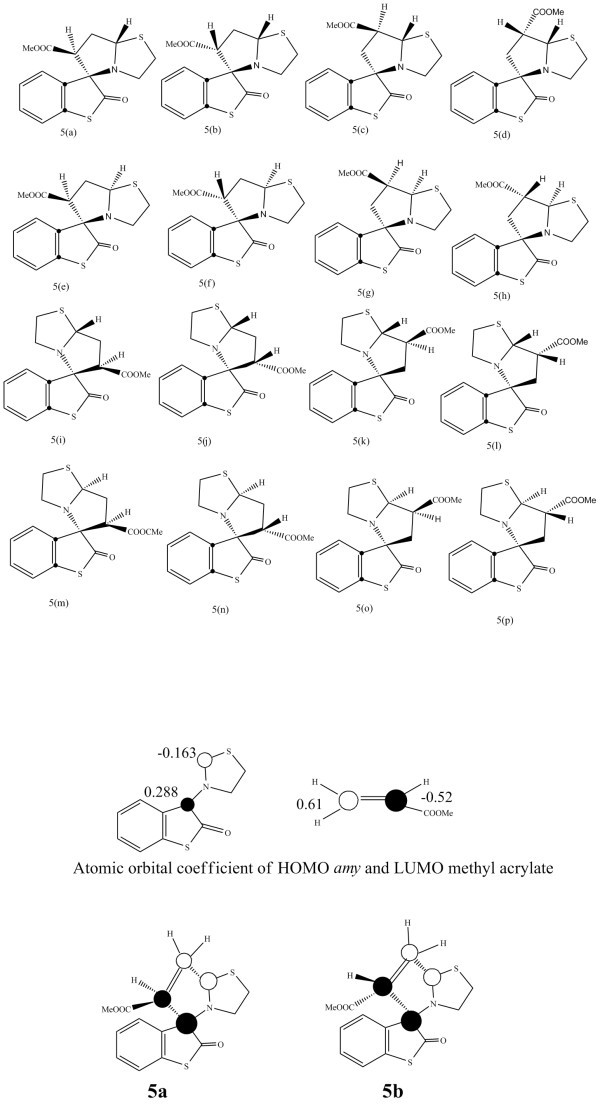
**Possible isomers of cycloadduct **5 **and its regioselectivity**.

Methyl acrylate may approach either of the *amy *with the formation of products having three chiral centers. Therefore a total 8 + 8 = 16 isomers **5a**-**5p **are possible (Figure 3).

Attack of methyl acrylate on *syn *amy results in the inward movement of thiazolidine ring towards thioisatin nucleus which imposes steric hindrance and makes it unstable. In fact we failed to locate the transition state in any such case **5i**-**5p**. The remaining 8 isomers may be obtained by the attack of methyl acrylate on *anti *amy. Out of these 8 possibilities only four **5a**-**5d **have concerted mechanism. We have optimized the geometries of all the four isomers. Results show that all isomers have almost same Δ*H*_f_, indicating that thermodynamically all are nearly equally stable.

**Figure 4:**
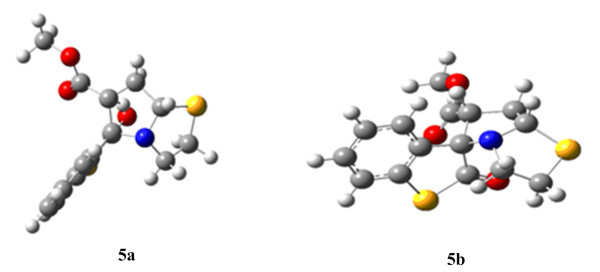
**Transition states of cycloadducts 5a and 5b**.

We have carried out transition state calculations on all the four isomers but have been successful in locating the transition state for only two isomers **5a**-**5b **(Figure 4).

The transition state of the concerted 1,3-dipolar cycloadditions is usually controlled by frontier molecular orbitals of dipolarophiles and dipole (azomethine ylide). The favoured path involves HOMO_dipole _and LUMO_dipolarophile_. The Δ*H*_f _, HOMO, LUMO energies and HOMO-LUMO energy gaps of azomethine ylides **4 **with dipolarphiles are given in Table 1.

**Table 1:** ΔH_f_, HOMO, LUMO, energies and H-L And L-H energy gaps

Cycloadduct	5a	5b	5c	5d
Δ*H*_f _(*Kcal/mol*)	81.34	82.98	82.86	82.68

From the Table, it may be concluded that HOMO_dipole_-LUMO_dipolarophile _energy gap is lower than the LUMO_dipole_-HOMO_dipolarophile _gap and therefore the dominant FMO approach is HOMO_dipole_-LUMO_dipolarophile_. Both the HOMO and the LUMO of the dipole show uneven distribution of the electron density along the C-N-C dipole. In the HOMO case, the orbital coefficient is larger at C_1 _(0.288) than at C_2 _(-0.163). Similarly in the LUMO of methyl acrylate the atomic orbital coefficient are (0.611) and (-0.521) respectively (Figure 3). Thus it may be concluded that there is better overlap when -COOMe group lies towards thioisatin ring, giving two possibilities **5a **and **5b**. Out of these **5a **is formed in diastereomeric excess probably due to the *endo *approach of -COOMe.

### 2.2 Regioselectivity of the addition of symmetrical and unsymmetrical acetylenes

**Figure 5:**
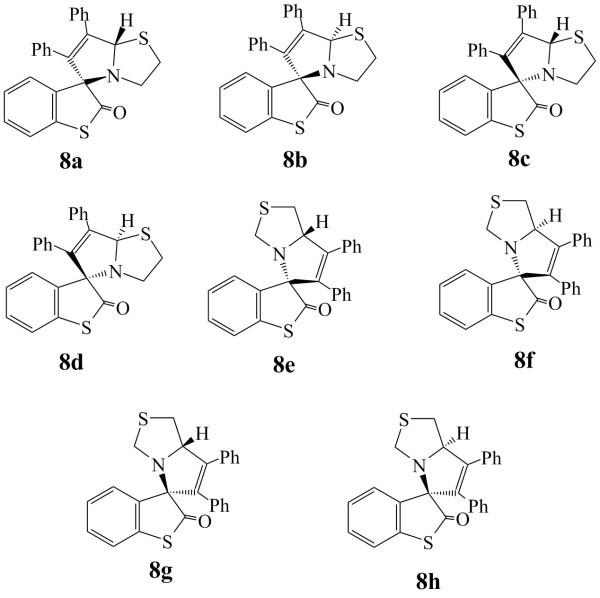
**Possible isomers of cycloadduct 8**.

Parallel to methyl acrylate, diphenylacetylene can also attack either of the azomethine ylide (**4*_syn _***or **4*_anti_***) with the formation of products having two chiral centers. Therefore a total of 4 + 4 = 8 isomers (4 pairs of enantiomers) could be possible **8a-8h **(Figure 5).

Attack of diphenylacetylene on *syn*-azomethine ylide **4*****_syn _***results in the inward movement of the thiazolidine ring towards the thioisatin nucleus and the transition state could not be located even in a single case (Figure 2) ruling out the possibility of the formation of products **8e-8h**. Thus it leaves the possibility of attack on the *anti *azomethine ylide **4*_anti _***and hence only four isomers **8a-8d **are left for consideration. We have optimized the geometry of all the four isomers. Results show that all isomers have almost same Δ*H*_f_, indicating that thermodynamically all are nearly equally stable.

**Figure 6:**
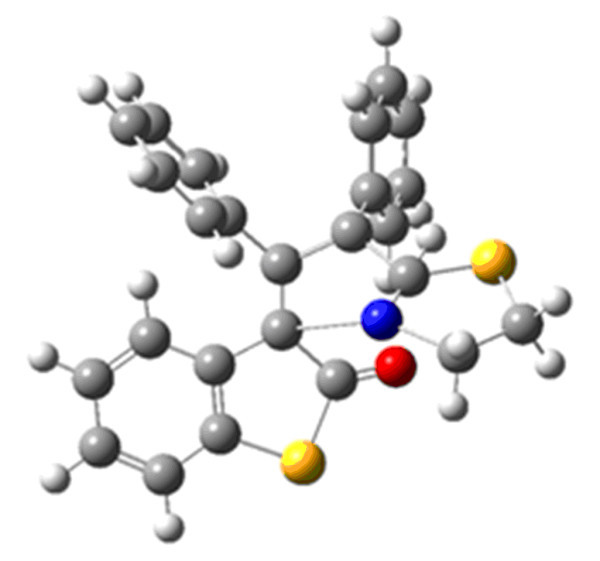
**Transition state of diphenylacetylene cycloadduct 8**.

**Figure 7:**
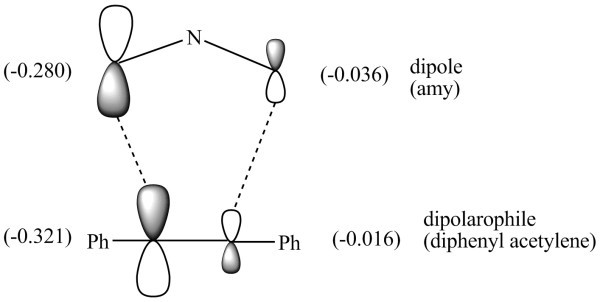
**Atomic orbital coefficients and overlapping of dipole(*amy*) with diphenylacetylene**.

Of remaining four possibilities **8c **and **8d **where N and H atoms on the adjacent carbon atoms do not lie on the same side, the transition state could not be located because concerted mechanism is not possible in such a situation. This leaves only two isomers **8a-8b **for consideration. Out of these two isomers we could optimize the transition state in case of **8a **only (Figure 6). This can be explained using the FMO approach along with the *endo *approach of the phenyl ring (Figure 7) as discussed above.

**Figure 8:**
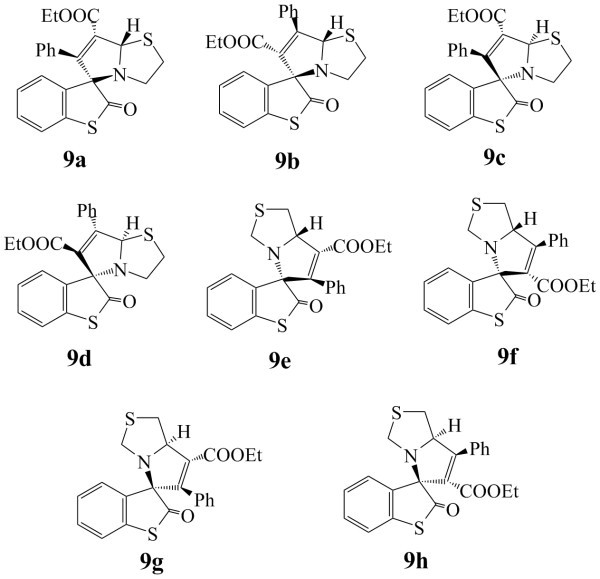
**Possible isomers of cycloadduct 9**.

**Figure 9:**
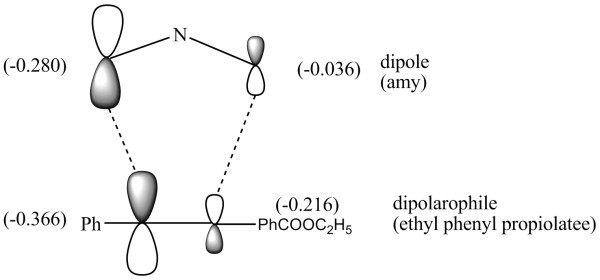
**Atomic orbital coefficients and overlapping of *amy *with ethylphenyl propiolate**.

**Figure 10:**
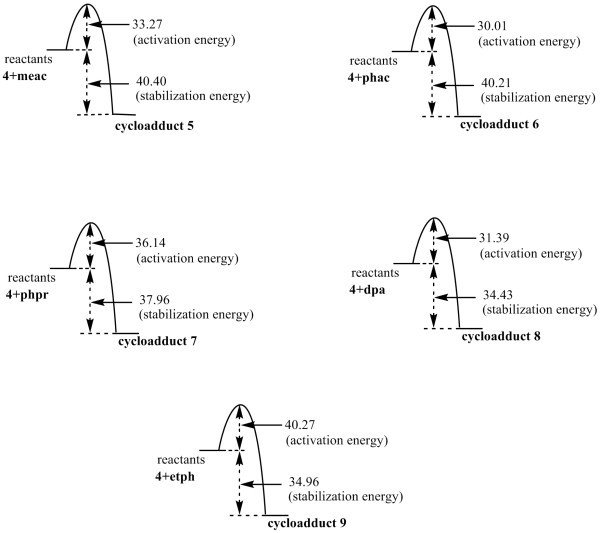
**Energy profile diagrams of azabicycloadducts **5-9 **(all values in Kcal/mol)**.

Similarly attack of unsymmetrical dipolarophile, such as ethyl phenyl propiolate, on *syn *or *anti *azomethine ylide may produce a cycloadduct having two chiral centres and therefore a total of 4 + 4 = 8 stereoisomers **9a-9h **could be possible (Figure 8) and it was concluded that isomer **9a **is formed preferentially (Figure 9). The energy profile diagrams for azabicycloadducts **5-9 **are presented in (Figure 10).

ΔH*_f_*_-R_, ΔH*_f_*_-Ts_, ΔH*_f_*_-P_, Ea(activation energy) and stabilization energy of amy with different dipolarophiles have been tabulated in Table 2.

**Table 2:** ΔH_f_-R, ΔH_f_-Ts, ΔH_f_-P, E*a*. and stabilization energy of a*my *with different dipolarophiles

Cycloadduct	8a	8b	8c	8d
ΔH_f _(*Kcal/mol*)	109.38	112.32	109.86	110.38

## 3. Conclusions

From the above discussions, it may be concluded that:

a.The azomethine ylide exits in two conformations; **4*****_syn _***and **4*_anti_***.

b. The dominant FMO approach is HOMO_dipole_-LUMO_dipolarophile _as this energy gap is lower than the LUMO_dipole_-HOMO_dipolarophile _gap.

c. Azomethine ylide is stabilized by the delocalization of dipolar charge into thioisatin nucleus, thus increasing the activation energy for the reaction path.

## 4. Experimental

The uncorrected melting points were taken in open glass capillaries. The IR spectra were recorded on a Nicolet Magna IR Spectrometer Model 550 in KBr pellets and band positions are reported in wave numbers (cm^-1^). The ^1^H NMR spectra and ^13^C NMR spectra have been recorded on a Bruker DRX-300 MHz and 75.47 MHz model respectively in CDCl_3 _and DMSO using tetramethylsilane as an internal standard. The chemical shifts are given in δ ppm values. The mass spectra were recorded on a JEOL-SX 102 (FAB). Most of the spectra were recorded at CDRI, Lucknow, India. Elemental analyses were performed on a Perkin Elmer Series C, H, N, S Analyzer 2400. The solvents were purified by standard procedures [18,19]. Acetonitrile was dried by refluxing with anhydrous calcium chloride for 5-6 h and then distilling it. Column chromatography was performed on silica gel 60 (Merck).

## Methods

### Synthesis of (5S,7R,8R)-spiro-{7-methoxycarbonyl-1-aza-4-thia-bicyclo [3, 3, 0]-octane 8, 3'}-benzo[b]thiophene-2'-one (5)

An equimolar mixture of thiosatin **1 **(0.36 gm, 2.0 mmol), thiazolidine-2-carboxylic acid **2 **(0.26 gm, 2.0 mmol) and methyl acrylate (0.32 gm, 2.0 mmol) in dry acetonitrile (50 ml) was refluxed for 22 h. The reaction was monitored by TLC until the consumption of the reactants. The reaction mixture was filtered, solvent evaporated and the residue was subjected to column chromatography. The petroleum ether/chloroform (4:1) fraction afforded the desired azabicycloadduct **5 **as pale brown solid.

Pale brown solid, yield: 0.33g (70%), mp: 105-107°C. IR (KBr): 1710(C = O),1450(C-N), 715(C-S) cm^-1^. ^1^H NMR (CDCl3, *δ *ppm): = 2.29 (1H, t, *J *= 3.0Hz 7-CH), 2.46(2H, dd, *J*_1 _= 4.2Hz, *J*_2 _= 3.3Hz 6-CH_2_), 2.50(2H, t, *J *= 1.2Hz 3-CH_2_), 2.68(2H, t, J = 2.1Hz 2-CH_2_), 3.10(3H, s, OCH_3_), 4.15(1H, t, *J *= 3.54 Hz, 5-CH), 7.36-7.54(4H, m, ArH). ^13^C NMR (CDCl3, *δ *ppm): 35.43(CH_2_S), 44.58(CH_2_), 46.34(CH), 85.99(C-N), 125.86-124.32(C = C), 142.58-131.36(CHaro), 175.67(O = C-O), 184.32(C = O). EI-MS: m/z (%) = 321(M+ 38), 275(100), 261(20), 284(15), 234(18). Anal. Calcd. For C_15_H_15_NO_3_S_2_: C, 56.05%; H, 4.70%; N, 4.36%. Found: C, 56.45%; H, 4.78%; N, 4.76%.

### (5R,8R)-spiro-{7-phenyl-1-aza-4-thia-bicyclo [3,3,0]-6-octene-8,3'}-benzo[b] thiophene-2'-one (6)

Coffee brown powder, yield: 0.31g (65%), mp: 95-97°C. IR (KBr): 1720(C = O), 1445(C-N), 690(C-S) cm^-1^. ^1^H NMR (CDCl3, *δ *ppm): = 2.50(2H, t, *J *= 3.0 Hz, 3-CH_2_), 2.68(2H, t, *J *= 3.3Hz, 2-CH_2_), 4.12(1H, d, *J *= 2.4Hz, 5-CH), 4.32(1H, d, *J *= 6.6Hz, 6-CH), 7.32-7.54(9H, m, ArH). ^13^C NMR (CDCl3, *δ *ppm): 31.51(CH_2_S), 36.54(CH_2_), 45.46(CH), 85.95(C-N), 128.81-124.49(C = C), 143.87-132.36(CHaro), 180.79(C = O). EI-MS: m/z (%) = 337(M+ 36), 203(100), 309(20), 108(34). Anal.Calcd for C_19_H_15_NOS_2_: C, 67.62%; H, 4.48%; N, 4.15%. Found: C, 67.78%; H, 4.53%; N, 4.32%.

### (5R,8R)-spiro-{7-phenyl-6-methyl-1-aza-4-thia-bicyclo [3,3,0]-6-octene-8,3'}- benzo[b]thiophene-2'-one (7)

Brownish solid, yield: 0.32g (68%), mp: 120-122°C. IR (KBr): 1725(C = O), 1420(C-N), 678(C-S) cm^-1^.^1^H NMR (CDCl3, *δ *ppm): 2.14(3H, d, *J *= 7.2 Hz, Me), 2.50(2H, t,*J *= 3.0Hz 3-CH_2_), 2.68(2H, t,*J *= 3.2Hz 2-CH_2_), 2.84(1H, q, *J *= 3.6Hz,5-CH), 7.59-8.45(9H, m, ArH). ^13^C NMR(CDCl3, *δ *ppm): 32.33(CH_2_S), 35.43(CH_2_), 44.45(CH), 86.54(C-N), 124.23-122.86(C = C), 143.41-130.63(CHaro), 184.34(C = O). Anal.Calcd for C_20_H_17_NOS_2_: C, 68.34%; H, 4.87%; N, 3.98%. Found: C, 68.55%; H, 4.93%; N, 4.13%.

### (5R,8R)-spiro-{6,7-diphenyl-1-aza-4-thia-bicyclo [3,3,0]-6-octene 8, 3'}- benzo[b]thiophene-2'-one (8)

Shiny brown powder, yield: 0.29g (63%), mp: 135-133°C. IR (KBr): 1715(C = O), 1420(C-N), 6905(C-S) cm^-1^. ^1^H NMR (CDCl3, *δ *ppm): 2.51(2H, t,*J *= 2.7Hz, 3-CH_2_), 2.60(2H, t, J = 3.0Hz, 2-CH_2_), 4.15(1H, s, 5-CH) 6.79-8.12(14H, m, ArH). ^13^C NMR (CDCl3, *δ *ppm): 34.54(CH_2_S), 45.43(CH_2_), 46.72(CH), 88.23(C-N), 127.32-126.54(C = C), 146.41-132.32(Caro), 183.54(C = O). EI-MS: m/z (%) = 413(M^+ ^32), 385(39), 235(100), 108(35). Anal. Calcd for C_25_H_19_NOS_2_: C, 72.61%; H, 4.63%; N, 3.39%. Found: C, 72.73%; H, 4.78%; N, 3.86%.

### (5R,8R)-spiro-{6-ethoxycarbonyl-7-phenyl-1-aza-4-thia-bicyclo [3,3,0]-6-octene-8, 3'}-benzo[b]thiophene-2'-one (9)

Dark brown power, yield: 0.31g (69%), mp: 110-112°C. IR (KBr): 1710(C = O), 1410(C-N), 690(C-S) cm^-1^. ^1^H NMR (CDCl3, *δ *ppm): 1.25(3H, t, *J *= 2.4Hz Me), 2.61(2H, t, *J *= 2.9Hz 3-CH_2_), 2.69(2H, t, *J *= 3.1Hz, 2-CH_2_), 2.67(2H, t, CH_2_), 3.75(2H, q, *J *= 6.3Hz, OCH_2_),4.12 (1H. s,5-CH) 7.49-7.94(4H, m, ArH). ^13^C NMR (CDCl_3_, *δ *ppm): 33.85(CH_2_), 44.96(CH), 46.32(CH_2_), 84.36(C-N), 124.32-122.93(C = C), 140.14-130.12(CHaro), 180.25(O = C-O), 185.44(C = O). EI-MS: m/z (%) = 321(M+ 38), 275(100), 261(20), 284(15), 234(18). Anal. Calcd for C_22_H_19_NO_3_S_2_: C, 64.52%; H, 4.68%; N, 3.42%. Found: C, 64.68%; H, 4.79%; N, 3.54%.

## Competing interests

The authors declare that they have no competing interests.
